# A Conundrum of r-Protein Stability: Unbalanced Stoichiometry of r-Proteins during Stationary Phase in Escherichia coli

**DOI:** 10.1128/mbio.01873-22

**Published:** 2022-08-18

**Authors:** Kaspar Reier, Petri-Jaan Lahtvee, Aivar Liiv, Jaanus Remme

**Affiliations:** a Institute of Molecular and Cell Biology, University of Tartu, Tartu, Estonia; b Department of Chemistry and Biotechnology, Tallinn University of Technology, Tallinn, Estonia; University of Würzburg

**Keywords:** proteomics, rRNA, ribosomal proteins, ribosomes, stationary phase

## Abstract

Bacterial ribosomes are composed of three rRNA and over 50 ribosomal protein (r-protein) molecules. r-proteins are essential for ribosome assembly and structural stability and also participate in almost all ribosome functions. Ribosomal components are present in stoichiometric amounts in the mature 70S ribosomes during exponential and early stationary growth phases. Ribosomes are degraded in stationary phase; however, the stability and fate of r-proteins during stationary growth phase are not known. In this study, we report a quantitative analysis of ribosomal components during extended stationary-phase growth in Escherichia coli. We show that (i) the quantity of ribosomes per cell mass decreases in stationary phase, (ii) 70S ribosomes contain r-proteins in stoichiometric amounts, (iii) 30S subunits are degraded faster than 50S subunits, (iv) the quantities of 21 r-proteins in the total proteome decrease during 14 days (short-lived r-proteins) concomitantly with the reduction of cellular RNA, and (e) 30 r-proteins are stable and form a pool of free r-proteins (stable r-proteins). Thus, r-proteins are present in nonstoichiometric amounts in the proteome of E. coli during the extended stationary phase.

## INTRODUCTION

Translation of genetic information into proteins occurs in ribosomes. Ribosome function is vital for life. Therefore, the quality and quantity of ribosomes in bacterial cells must meet the requirements for protein production during adaptation to changing environmental conditions. Ribosome biogenesis is most active during early log phase when protein synthesis is at its maximum and then gradually decreases (1). A single Escherichia coli cell contains up to 70,000 ribosomes during exponential growth on glucose and up to 10 times fewer after the cessation of growth (1). The ribosome content in bacterial cells is tightly controlled by several regulatory mechanisms during the progression of the growth phase (2–4).

Bacterial ribosomes are composed of three rRNA chains and over 50 protein (r-protein) molecules. Proteins make up about one-third of the ribosome by mass, and the remaining two-thirds is rRNA. rRNA and r-proteins are organized into two unequal subunits. In E. coli, the small ribosomal subunit (30S) consists of 16S rRNA (1,543 nucleotides) and 21 r-proteins (bS1 to bS21). The large ribosomal subunit (50S) comprises 23S rRNA (2,904 nt), 5S rRNA (120 nt), and 33 different protein chains (bL1 to bL36). All components are present in a single copy in the mature ribosome (70S) except for the protein bL7/bL12, which is associated with the ribosomes as a tetramer via the uL10 protein (5, 6). The majority of the r-proteins are small and basic. They are important for ribosome assembly and for maintaining ribosome structure and function (6).

Translation initiation starts with the 30S subunit binding the initiator tRNA and mRNA in its decoding center (DC) (7). DC is responsible for the recognition of the appropriate aminoacyl-tRNA (aa-tRNA) anticodon guided by the mRNA codons during the elongation phase of translation (7). The 50S subunit encloses the peptidyl transferase center (PTC), which catalyzes the formation of peptide bonds in the nascent protein chain during elongation, as well as the release of the full-length protein at the end of translation (7, 8). The ribosome functional centers DC and PTC are formed of both rRNA and r-proteins (7, 8). Chemical or enzymatic damage to ribosomal components can compromise ribosome functioning (9). Partially active ribosomes must either be degraded or repaired to restore their full functionality (9, 10).

The number of ribosomes per cell starts to decrease during late log phase due to two processes (11). First is the dilution effect: ribosome synthesis slows significantly while cell division continues. Second, ribosomes are degraded in cells as demonstrated by monitoring rRNA degradation (4). rRNA degradation has been shown to occur under stress conditions, e.g., under inhibition of translation in the presence of antibiotics (12), upon rRNA overproduction (13, 14), and upon entry into stationary phase (15).

While rRNA degradation is well documented (4, 16), the fate of r-proteins in stationary phase has remained unknown (17, 18). Here, we aim to fill this gap by analyzing the r-protein quantities in the ribosomes, as well as in the whole-cell proteome, during stationary-phase growth of E. coli. We use quantitative mass spectrometry to measure the stability and composition of r-proteins during stationary-phase growth of E. coli cultures. We show that ribosomes contain nearly all r-proteins in equimolar amounts until late stationary phase; however, the degrees of stability of individual r-proteins in the proteome are different. Surprisingly, it appears that about two-thirds of 51 of the r-proteins are stable during stationary-phase growth over the course of 14 days, while the remaining r-proteins are degraded concomitantly with the rRNA. Thus, r-proteins in the E. coli proteome are nonstoichiometric during extended stationary-phase growth.

## RESULTS

### Experimental setup.

**FIG 1:**
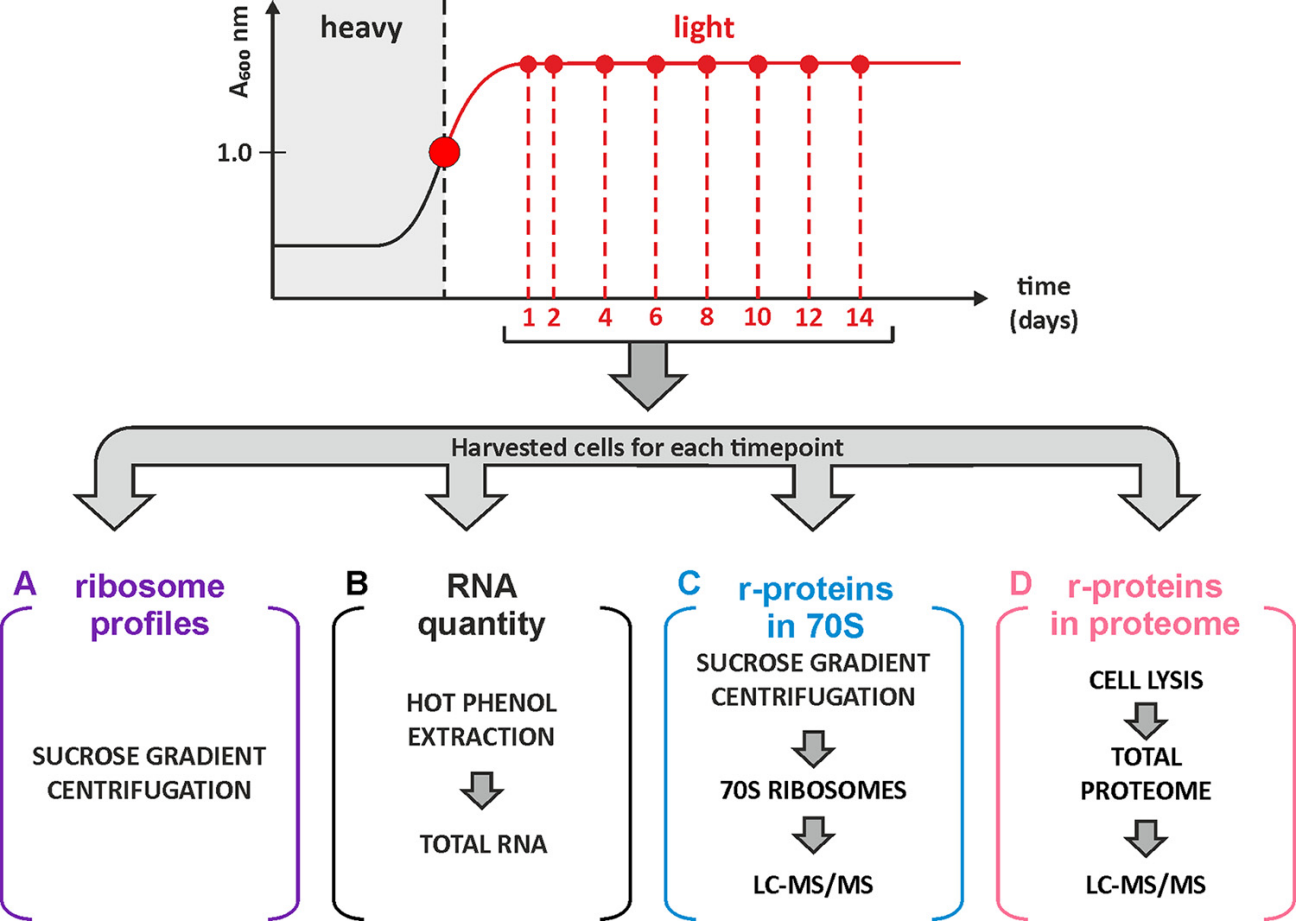
Experimental design. E. coli strain MG1655-SILAC was grown in medium containing heavy-labeled arginine and lysine to mid-log phase, followed by a chase with a 20-fold excess of unlabeled (light) arginine and lysine, and grown for 2 weeks to obtain stationary-phase cells. Samples were collected at the indicated time points and divided into three aliquots. Then, ribosomes were isolated using sucrose gradient centrifugation (A), the RNA quantity was determined by hot-phenol extraction (B), the r-protein quantity was determined using LC-MS/MS (C), and the r-protein stoichiometry in the total proteome was ascertained using LC-MS/MS (D).

The aim of this study was to determine the fate of ribosomes and r-proteins. For this, a SILAC (stable isotope-labeled amino acids in cell culture)-based experimental approach was used (Fig. 1). E. coli cells were grown in MOPS (morpholinepropanesulfonic acid) medium supplemented with “heavy”-labeled arginine (Arg10, [^13^C]_6_H_14_[^15^N]_4_O_2_) and lysine (Lys8, [^13^C]_6_H_14_[^15^N]_2_O_2_). At mid-log phase, the culture was further supplemented with a 20-fold molar excess of “light” unlabeled arginine (Arg0) and lysine (Lys0), divided into 8 aliquots, and grown for 14 days. Cell samples were collected at day 1 (24 h) and day 2 (48 h) and subsequently at 48-h intervals over the following 12 days. Bacterial cell viability analysis revealed that the number of viable cells (CFU) was highest on day 2 and subsequently decreased modestly (Fig. S1 posted at http://dx.doi.org/10.23673/re-310). At each sampling point, four different data sets were generated. First, the ribosome particle distribution was analyzed by sucrose gradient centrifugation. Peak areas of the 70S and free ribosomal subunit fractions were quantified, and 50S/70S or 30S/70S ratios were calculated. Second, the rRNA amounts in the samples were estimated, assuming that 80% of the total RNA of the bacterial cell corresponds to the rRNA (1, 19). Total RNA was isolated from cells using hot-phenol extraction, the concentration was measured, and values were normalized against the value for day 1. Third and fourth, the quantities of r-proteins in the 70S ribosome fraction and the total proteome were determined using SILAC-based liquid chromatography-tandem mass spectrometry (LC-MS/MS) and normalized to the corresponding values from day 1. The latter allowed us to directly distinguish r-protein degradation in ribosomes versus in the whole proteome, which included the amount of nonbound (“free”) r-proteins.

### Unequal accumulation of ribosome subunits in stationary phase.

**FIG 2:**
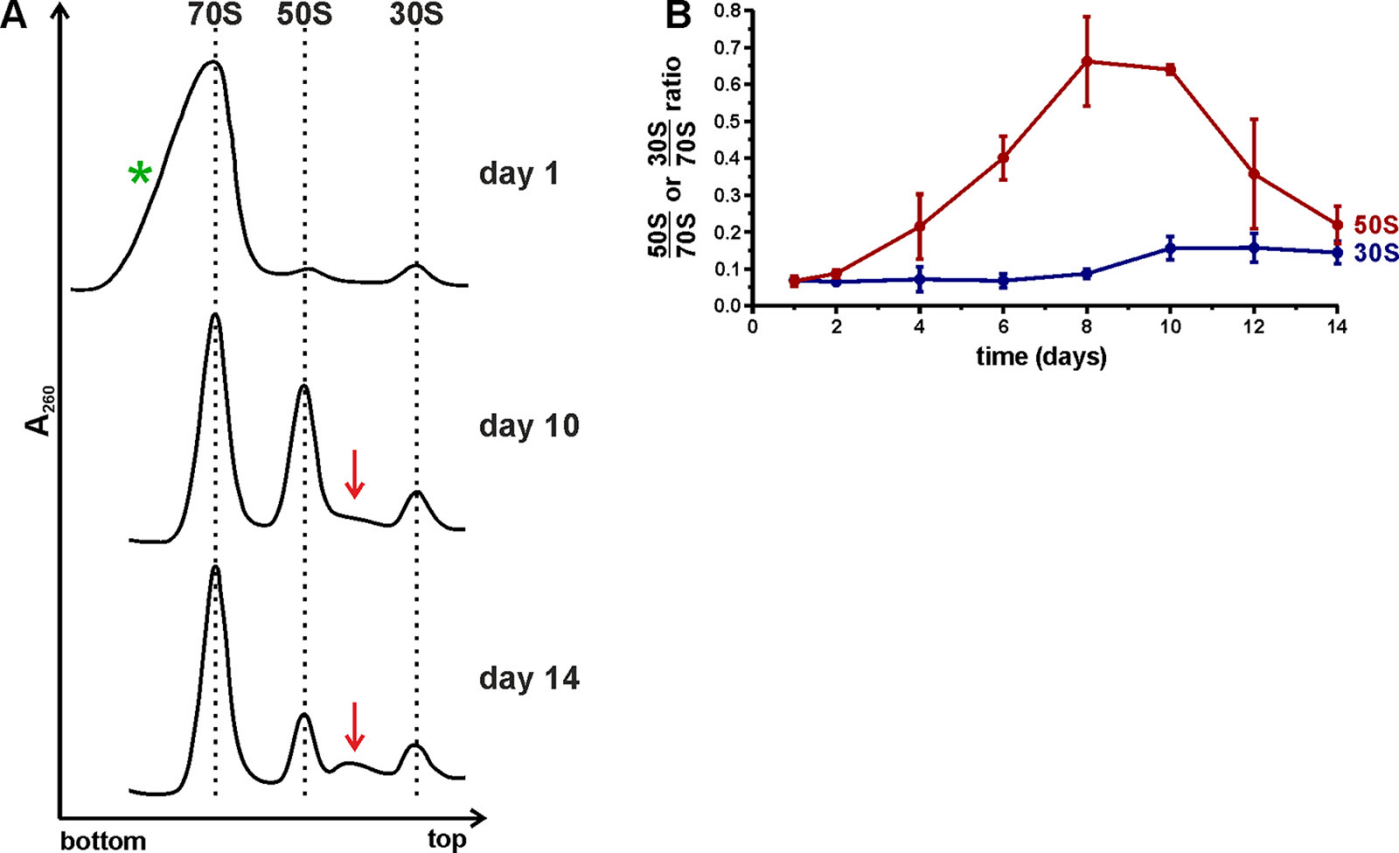
Ribosome profile analysis of stationary-phase cells using sucrose gradient centrifugation. Cells were harvested from stationary-phase cultures over the course of 14 days. (A) Representative ribosome profiles from day 1, day 10, and day 14. The direction of sedimentation is from right to left. Red arrows show the accumulation of intermediate particles. The green asterisk indicates a notable shoulder present on the 70S peak at the beginning of stationary phase. The complete data set is shown in Fig. S2 at http://dx.doi.org/10.23673/re-310. The ribosome patterns are normalized to the 70S peak. (B) Free subunit-to-70S ribosome ratios calculated based on the corresponding ribosome profiles. Areas under the 70S, 50S, and 30S peaks were quantified using ImageJ, and the subunit/70S ratios were calculated and plotted (*y* axis). Values shown in the figure are the mean results from three independent biological experiments with standard deviations (*n* = 3; mean ± SD).

To find out how the ribosome population changed during stationary phase, the ribosome contents in batch culture over the course of 14 days were analyzed by sucrose gradient centrifugation (Fig. 1A). Representative ribosome profiles from day 1, day 10, and day 14 are shown in Fig. 2A (for a complete set of ribosome profiles, see Fig. S2 at http://dx.doi.org/10.23673/re-310). The 70S peaks from days 1 to 4 exhibited a notable shoulder (Fig. 2A; Fig. S2 at the URL mentioned above) that was identified as 100S particles according to sucrose gradient analysis using specific conditions as specified in the legend to Fig. S3 at the URL mentioned above. To quantify how the free ribosomal subunit amounts changed in comparison to each other, the ratios between peak areas of free subunits and 70S ribosomes (50S/70S and 30S/70S ratios, respectively) were calculated from the gradient profiles (Fig. 2B). Finally, the results were controlled for multiple comparisons using the Bonferroni method (Table S1 at the URL mentioned above).

On day 1, the 50S/70S and 30S/70S ratios were ≈0.07, indicative of low levels of free subunits present in the cells, in agreement with published data (20). By day 4, the 50S/70S ratio was ≈0.21, and it increased further up to ≈0.64 by day 10, indicating the accumulation of free 50S subunits. In contrast, the 30S/70S ratios remained low over the course of 14 days. After day 10, the 50S/70S ratios decreased down to ≈0.19, whereas the 30S/70S ratio remained stable. It is worth noting that after 10 days, particles sedimenting between the 50S and 30S subunits started to accumulate (Fig. 2A; Fig. S2 at http://dx.doi.org/10.23673/re-310). These 40S-like particles were probably degradation intermediates of the 50S subunits. The accumulation and delayed reduction of free 50S subunits indicated a slower degradation of 50S subunits than of 30S subunits in stationary phase.

### Total RNA content decreases in stationary phase.

**FIG 3:**
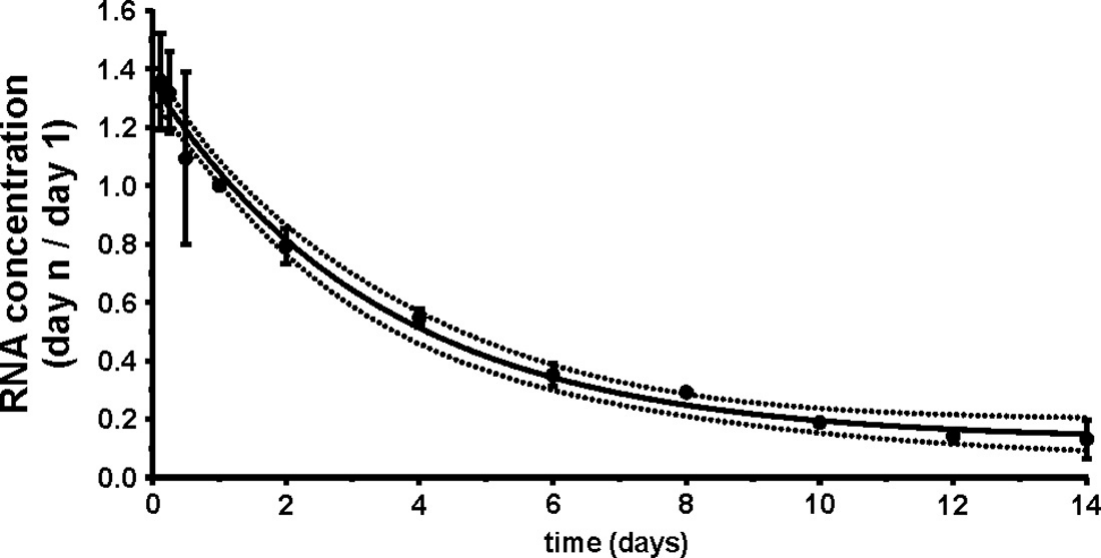
Total RNA content in cells decreases during the stationary phase. Samples were collected over the course of 14 days. Total RNA was extracted from cells using hot-phenol extraction and ethanol precipitation. The RNA concentration was determined by measuring absorbance at 260 nm and normalized to the corresponding value on day 1 (*y* axis). Data were fitted into a one-phase decay model, where the solid line represents the mean values and the dotted lines the 95% confidence intervals. The values shown in the figure are the mean results from three independent biological experiments with standard deviations (*n* = 3; mean ± SD).

Ribosomes are known to be stable in growing bacteria (21), however, as cells enter stationary phase, the ribosome amount per cell decreases (15). Based on previous reports that rRNA makes up 80% of the total RNA in cells (1, 19), the rRNA content can be evaluated by quantifying total RNA in cells. To determine the relative quantities of rRNA during stationary phase, the concentrations of total RNA in stationary-phase cells were measured over the course of 14 days. Total RNA was extracted from 2 mg of wet cell mass, and the RNA concentration was determined and compared with the measurement from day 1. Finally, the data were fitted onto a one-phase decay model (Fig. 3) and controlled for multiple comparisons using the Bonferroni method (Table S2 at http://dx.doi.org/10.23673/re-310). As a control, the amount of total RNA was determined before cells entered stationary phase (3, 6, and 12 h after the start of the growth). The total RNA concentration per cell mass started to decrease at late log phase (Fig. S4 at the URL mentioned above), continued to decrease with the progression of the stationary phase, and reached an apparent plateau on day 6 (Fig. 3). After day 6, no statistically significant decrease in RNA concentration was detected. At day 6, the amount of RNA was decreased by 65% compared to the amount at day 1 (Fig. 3). 16S and 23S rRNA were quantified in total RNA by dot-plot hybridization (Fig. S5 at the URL mentioned above). The fractions of both 16S and 23S rRNA decreased over time; however, the decrease of the 23S rRNA fraction was significantly slower than that of 16S rRNA during days 6 to 10. This was reminiscent of the free ribosome subunit content (compare Fig. 2B; Fig. S5C at the URL mentioned above). In conclusion, the decrease of total RNA content in the cells was attributed to the degradation of rRNA in the cells.

### Stationary-phase ribosomes contain equimolar amounts of r-proteins.

**FIG 4:**
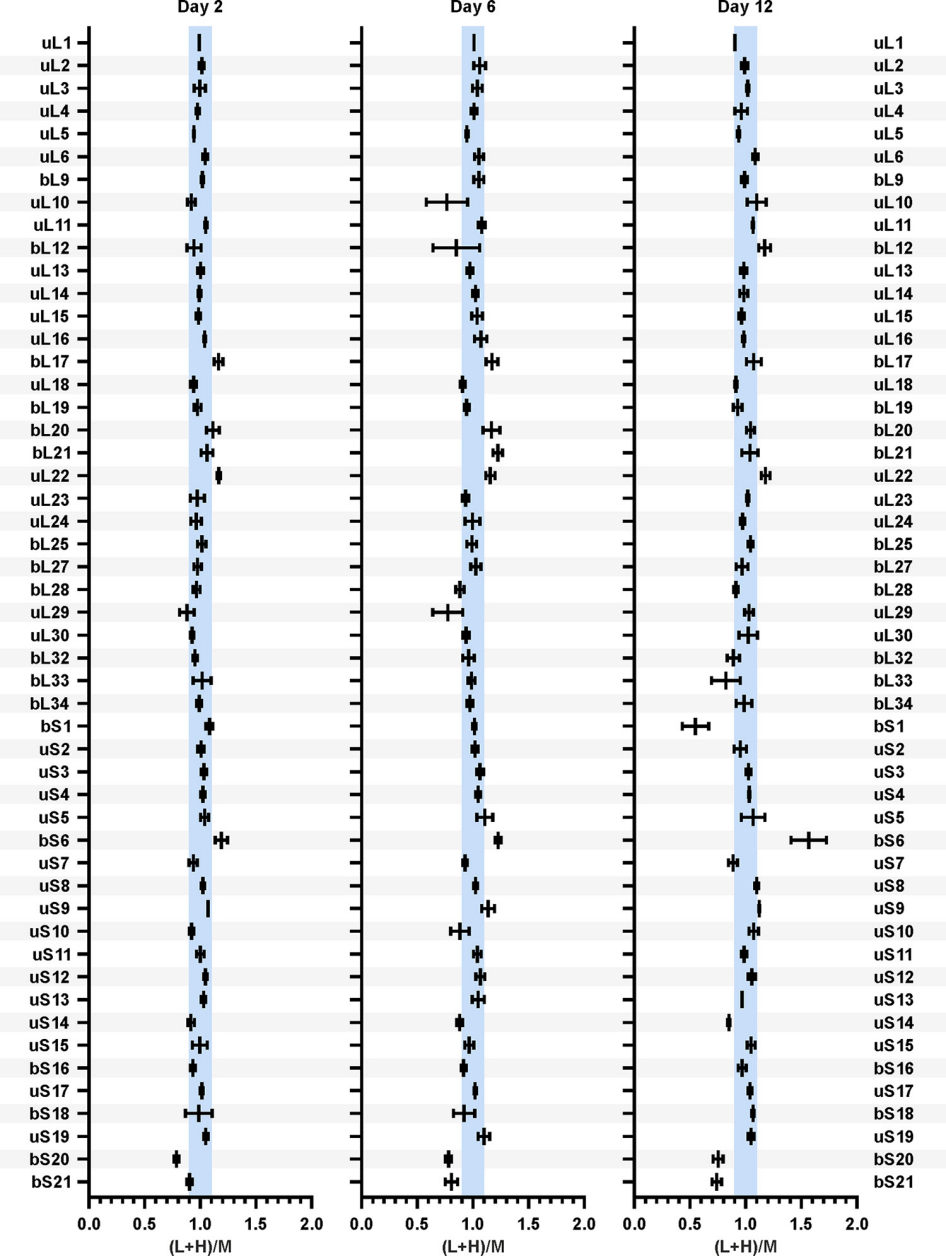
r-protein quantities in the 70S ribosomes during the stationary phase. Cells were collected over the course of 14 days. 70S ribosomes were isolated and mixed in a 1:1 ratio with medium-heavy-labeled reference 70S ribosomes for r-protein quantification using LC-MS/MS. Proteins bL31A, bL31B, bL35, bL36A, and bL36B were not quantified due to insufficient numbers of unique peptides. Data sets from days 2, 6, and 12 are shown, and the complete set of all data sets is shown in Fig. S6 at http://dx.doi.org/10.23673/re-310. The relative quantities of 51 r-proteins are presented as the (L+H)/M ratios (L+H = sample; M = reference). 50S r-protein (L+H)/M ratios were normalized against the average (L+H)/M ratio of all 50S r-proteins. 30S r-protein (L+H)/M ratios were normalized against the average (L+H)/M ratio of all 30S r-proteins. The blue-shaded areas mark the ±10% range of (L+H)/M ratios. Values shown in the figure are the mean results from three independent biological experiments with standard deviations (*n* = 3; mean ± SD).

In our previous study, we showed that the ribosome r-protein composition did not change during the transition of the cell culture from the exponential to the stationary phase except for bL31 and bL36 paralogues (22). Also, it has been shown that ribosomes contain stoichiometric amounts of r-proteins during exponential growth (5, 6, 23). In the present study, we focused on the changes in r-protein stoichiometry in the ribosomes during the stationary phase. 70S ribosomes were isolated from stationary-phase cells and mixed in a 1:1 molar ratio with the internal reference, a “medium-heavy”-labeled (Arg6, [^13^C]_6_H_14_N_4_O_2_, and Lys4, C_6_H_10_[^2^H]_4_N_2_O_2_) 70S ribosome isolated from the mid-log growth phase. The addition of the internal reference enabled the comparison of individual r-protein stoichiometries in ribosomes at different time points. Proteins were digested into peptides and analyzed using LC-MS/MS. Based on the MS results, the relative quantities of 51 r-proteins in the ribosomes were ascertained, while proteins bL31A, bL31B, bL35, bL36A, and bL36B were not quantified due to insufficient numbers of unique peptides. Finally, the results were controlled for multiple comparisons using the Bonferroni method, and only statistically significant results are discussed.

r-protein quantities in the 70S ribosomes from stationary-phase bacteria are presented as the (L+H)/M ratios (Fig. 4; complete data set is shown in Fig. S6 and statistical analysis in Table S3 at http://dx.doi.org/10.23673/re-310). The (L+H)/M ratio compares the relative quantity of an r-protein in a stationary-phase sample (L+H) to the quantity in the reference ribosome (M). 50S r-protein (L+H)/M ratios are normalized to the average (L+H)/M ratio of all 50S r-proteins. Similarly, 30S r-protein (L+H)/M ratios are normalized to the average (L+H)/M ratio of all 30S r-proteins. For 43 of the analyzed r-proteins, the (L+H)/M ratio was 1 ± 10%, meaning that these r-proteins were present in stoichiometric amounts (Fig. 4; Fig. S6 and Table S3 at the URL mentioned above). However, for proteins bS1 and bS21, the (L+H)/M ratios decreased over time. bS1 has previously been found in nonstoichiometric amounts on 70S ribosomes (24–26). bS20 exhibited an (L+H)/M ratio of 0.75 throughout stationary-phase growth. This systematic difference was likely caused by the unbalanced bS20 composition of the medium-heavy-labeled reference ribosomes. In the case of bL7/bL12 and uL10, the (L+H)/M ratios were highly variable (Fig. 4; Fig. S6 and Table S3 at the URL mentioned above). It has been shown that bL7/bL12 and uL10 are loosely bound to the ribosome (27), which explains the variability arising from the partial loss of these proteins during ribosome isolation. In the case of proteins bL17, uL22, and bS6, the (L+H)/M ratios increased during stationary-phase growth. However, when isolated 70S ribosomes were further disassociated into 50S and 30S subunits, the stoichiometry of these proteins was restored (Fig. S7 at the URL mentioned above). This increase could be attributed to nonspecific binding of bL17, uL22, and bS6 to the 70S ribosomes during isolation (Fig. S7 at the URL mentioned above). The loss of proteins bS1 and bS21 demonstrated another level of ribosome heterogeneity during stationary phase, in addition to the replacement of paralogous proteins bL31A and bL36A by their respective B paralogs (22). Taken together, for a majority of the r-proteins, their stoichiometry remained constantly unchanged in the 70S ribosomes during stationary phase.

### r-proteins are present in stoichiometric amounts in the early-stationary-phase proteome.

**FIG 5:**
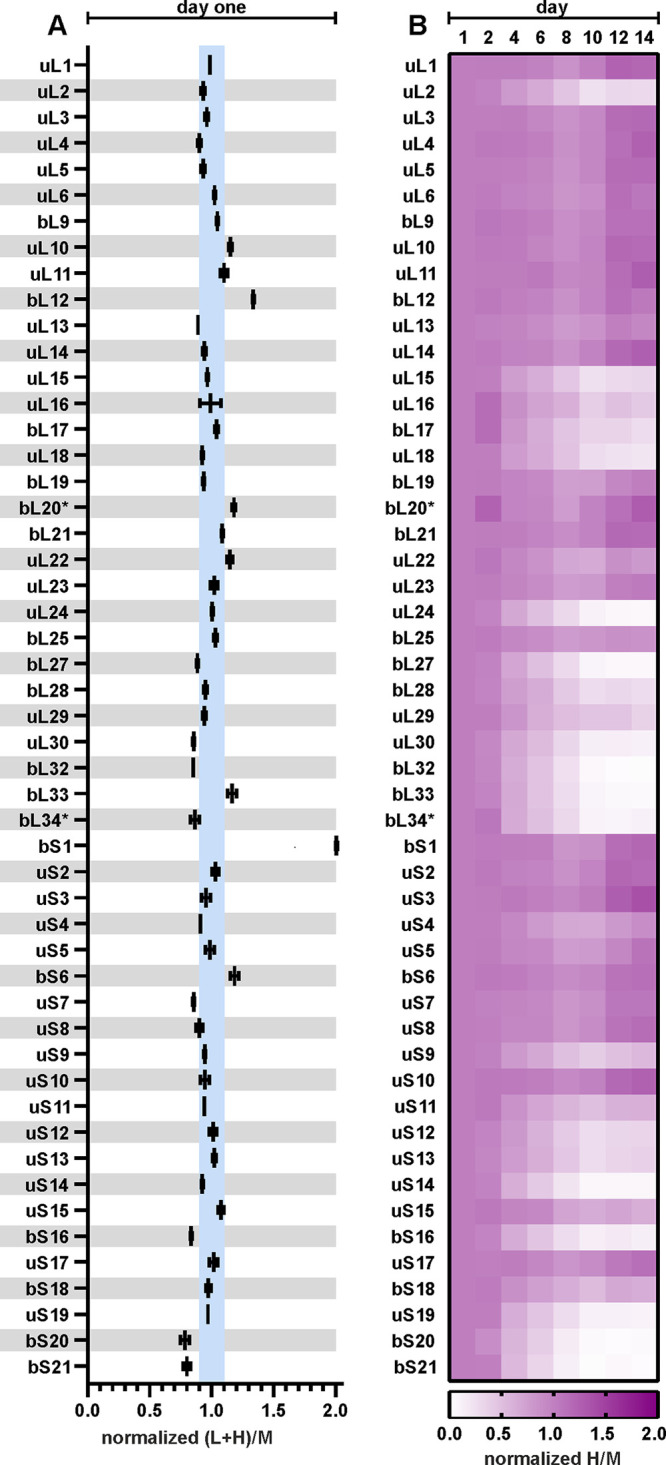
r-proteins become progressively nonstoichiometric during stationary phase. Cells were collected over the course of 14 days and lysed, and total protein was mixed in a 1:1 ratio with medium-heavy-labeled total protein from the reference cells for r-protein quantification using LC-MS/MS. Proteins bL31A, bL31B, bL35, bL36A, and bL36B were not quantified due to insufficient numbers of unique peptides. (A) Day 1 r-protein stoichiometry. r-protein relative quantities are presented as the (L+H)/M ratios (L+H, sample; M, reference). 50S r-protein (L+H)/M ratios were normalized against the average (L+H)/M ratio of all 50S r-proteins. 30S r-protein (L+H)/M ratios were normalized against the average (L+H)/M ratio of all 30S r-proteins. The blue-shaded areas mark the ±10% range of (L+H)/M ratios. Values shown in the figure are the mean results from two independent biological experiments with standard deviations (*n* = 2; mean ± SD). (B) Heatmap representing r-protein stoichiometry in the total proteome over the 14 days of stationary-phase growth. The normalized H/M ratios describe the relative quantities of r-proteins compared to the quantities on day 1. Proteins marked with an asterisk (*) had only one quantified peptide in their data sets. Values shown in the figure are the median results from three independent biological experiments (*n* = 3; median).

To shed a light on the stoichiometry and stability of r-proteins during prolonged stationary phase, we first performed a quantitative analysis of the early-stationary-phase proteome (day 1). The stoichiometry of individual r-proteins in the total proteome on day 1 was compared to that of r-proteins in the reference 70S ribosomes (Fig. 5A; Table S4 at http://dx.doi.org/10.23673/re-310). Total protein from day 1 cells was mixed in a 24:1 mass ratio with the medium-heavy-labeled 70S ribosomes and analyzed as described above. Forty-eight of the 51 detected r-proteins exhibited an (L+H)/M ratio of approximately 1 (±10%). This indicated that they were present in the total proteome of early-stationary-phase cells in the same stoichiometry as in the ribosomes. r-proteins bL7/bL12 and bS1 had (L+H)/M ratios of 1.3 and 2.0, respectively (Fig. 5A; statistical analysis is in Table S4 at the URL mentioned above). This meant that there was 30% more bL7/bL12 and two times more bS1 in the cells than in the ribosomes. This was in good agreement with earlier data (28, 29), demonstrating that these proteins had significant free pools *in vivo*. Given that according to our data, most of the r-proteins were present in the same stoichiometry in the early-stationary-phase total proteome as in the ribosomes, the day 1 proteome data set was further used as a reference.

### r-protein stoichiometry in the total proteome changes during stationary phase.

As we had demonstrated that r-proteins were stoichiometric in early stationary phase, we next wanted to determine if this stoichiometry changed during stationary phase. For that, we analyzed the r-protein quantities in the total proteome during the course of 14 days. The H/M and L/M ratios describe r-protein subpopulations that are synthesized in early log phase (H/M) or in late log phase and after the entry to stationary phase (L/M). Changes in the 51 r-protein H/M ratios are summarized in the heatmap in Fig. 5B. For 30 r-proteins (uL1, uL3, uL4, uL5, uL6, bL7/bL12, bL9, uL10, uL11, uL13, uL14, bL19, bL20, bL21, uL22, uL23, bL25, bS1, uS2, uS3, uS4, uS5, bS6, uS7, uS8, uS10, uS11, uS15, uS17, and bS18), the H/M (Fig. 5B) and L/M (Fig. S10 at http://dx.doi.org/10.23673/re-310) ratios in the total proteome did not change. Hence, their relative quantities per total protein mass remained the same as on day 1 (Fig. 5B; Fig. S8 to S10 and Table S5 at the URL mentioned above). Since these r-proteins showed no changes in measured quantities, they were considered stable r-proteins. On the other hand, for 21 r-proteins (uL2, uL15, uL16, bL17, uL18, uL24, bL27, bL28, uL29, uL30, bL32, bL33, bL34, uS9, uS12, uS13, uS14, bS16, uS19, bS20, and bS21), the H/M (Fig. 5B) and L/M (Fig. S10 at the URL mentioned above) ratios decreased over the next 14 days, meaning they were degraded (Fig. S8 and S9 and Table S5 at the URL mentioned above). As their measured quantities decreased in stationary phase, they were named short lived. It is important to note that both bS1 and bS21 disassociated from ribosomes, but while bS1 remained present in the total proteome (stable), bS21 was rapidly degraded (short lived). In contrast to the r-protein stoichiometry in the ribosome (Fig. 4; Fig. S6 at the URL mentioned above), the r-proteins were found in nonstoichiometric amounts in the total cell proteome. In conclusion, according to stationary-phase proteome data, the r-proteins could be divided into two distinct groups containing (i) 30 stable r-proteins and (ii) 21 short-lived r-proteins.

### r-protein degradation rates are disparate from each other.

**FIG 6:**
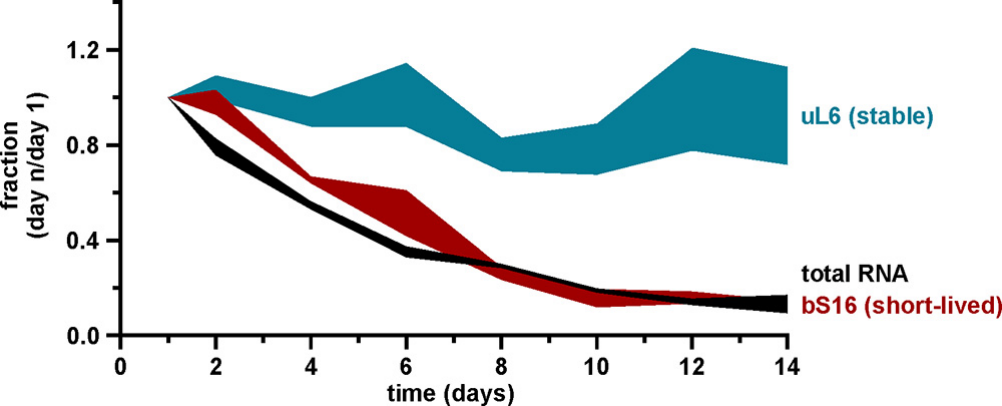
Cellular content of total RNA and r-proteins in stationary phase. Data sets showing quantities of total RNA (Fig. 3) and r-proteins uL6 and bS16 (Fig. S6 and S7 at http://dx.doi.org/10.23673/re-310) in stationary phase were compared to each other. Quantity is represented as the fraction of the value from day 1 during stationary phase (*y* axis). Values shown in the figure are the mean results from three independent biological experiments with standard deviations (*n* = 3; mean ± SD).

**FIG 7:**
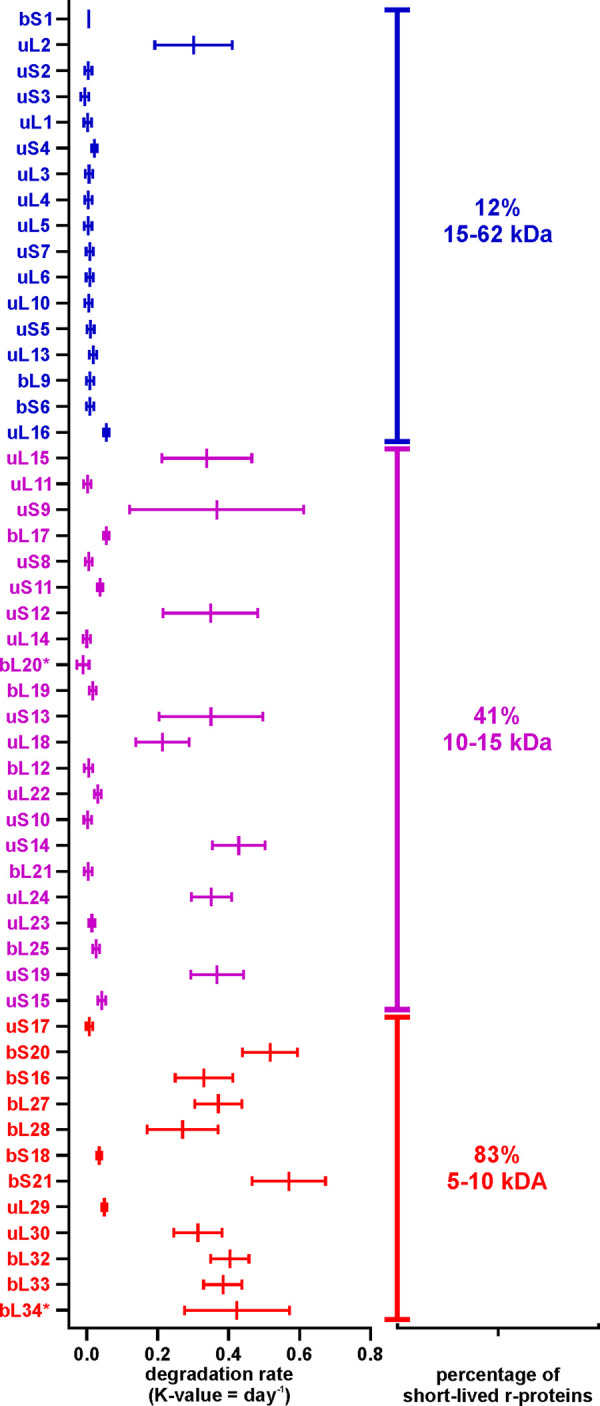
The degradation rate of an r-protein is related to its molecular weight (MW). The r-protein degradation rates were determined using linear regression analysis. *x* axis, degradation rates (*k* value = day^−1^); *y* axis, r-proteins sorted by molecular weight (from top to bottom). Values shown in the figure are the mean results from three independent biological experiments with standard deviations (*n* = 3; mean ± SD).

To quantitatively evaluate the r-protein degradation via the degradation rates, the r-protein dynamics in the stationary phase were modeled using nonlinear regression analysis. The r-protein quantities in the course of 14 days were fitted into the following models: (i) an exponential model, with a plateau followed by one-phase decay, and (ii) a linear model, a straight line. The fit between the data and the two models was evaluated using Akaike’s information criterion (30). Two representative examples, one for stable (uL6) and one for short-lived (bS16) proteins, are presented in Fig. 6 (for the complete data set, see Fig. 3; also see Fig. S8 and S9 at the URL mentioned above). The quantity of uL6 did not change during 14 days, following the linear model (stable protein), while the quantity of bS16 fit the exponential model (short-lived protein). For each r-protein, a degradation rate constant was calculated (*k* value for the exponential model and slope for the linear model) (Fig. 7; Table S6 at the URL mentioned above). These values characterized how fast the relative amounts of the specific protein changed in time. The degradation rates of the r-proteins were compared with their mass (Fig. 7). r-proteins with molecular masses of over 15 kDa were stable, except for uL2, while half of the smaller r-proteins were short lived. Moreover, the degradation trendlines of short-lived proteins tended to coincide with the RNA degradation curve during stationary phase (Fig. 6). At the same time, stable r-protein quantities remained almost unchanged in the total proteome during day 1 to day 14.

To sum up, the quantities of stable proteins could be fitted into the straight-line model, while the quantities of short-lived proteins could be characterized by the plateau followed by one-phase decay model (Fig. 6; Fig. S11 and S12 and Table S6 at the URL mentioned above). Accordingly, a specific set of r-proteins was not degraded concomitantly with the rRNA, instead forming a free pool of r-proteins in the cell (Table S7 at the URL mentioned above).

## DISCUSSION

In this paper, the stability of ribosomes and their components in E. coli during extended stationary-phase growth is determined. The ribosome gradient profiles reveal an accumulation of free 50S subunits, while no significant accumulation of free 30S subunits is detected during extended stationary-phase growth (Fig. 2B; Fig. S2 at http://dx.doi.org/10.23673/re-310). Therefore, free 30S subunits are degraded faster than free 50S subunits, leading to a nonstoichiometric ribosome subunit content during stationary phase. In accordance, 16S rRNA is degraded faster than 23S rRNA (Fig. S5 at the URL mentioned above). However, the decay of 30S subunit rRNA cannot solely account for the loss of total RNA during the first 6 days of stationary-phase growth (Fig. 3; Fig. S5 at the URL mentioned above). This is based on the observation that the total RNA content is reduced by 70% but the 30S subunit rRNA constitutes only 30% of the total cellular RNA (1). Therefore, a significant amount of the 50S subunit rRNA (23S and 5S rRNA) must be turned over as well. Indeed, degradation of 16S and 23S rRNA is evident from hybridization experiments (Fig. S5 at the URL mentioned above). Again, 23S rRNA appears to be more stable, leading to nonstoichiometry of 16S versus 23S rRNA. Ribosome degradation has been suggested to start with the dissociation of ribosome subunits (4), an idea that is supported by the results presented here. An excess of free 30S subunits can lead to nonproductive 30S initiation complex formation with initiation factors, initiator tRNA, and mRNA, thereby sequestering mRNA. This in turn can impede translation. Preferential degradation of 30S subunits is likely a failsafe mechanism to avoid the binding and trapping of mRNA molecules in the 30S initiation complexes. We suggest that the degradation of ribosomal subunits likely proceeds via distinct pathways, which is supported by previous studies (12, 31). The delayed degradation of free 50S subunits supports the vehement degradation of free 30S subunits (Fig. 2B; Fig. S2 at the URL mentioned above). Slower degradation of the 50S subunits is further stressed by the accumulation of particles sedimenting between the 50S and 30S fractions during extended stationary-phase growth (Fig. 2A, red arrow). In line with the ribosome subunit stability is the fact that 23S rRNA is more stable than 16S rRNA (Fig. S5 at the URL mentioned above). Considering that synthesis of new ribosomes is negligible during late stationary phase (32), these particles are likely the degradation products of 50S subunits. The fact that no 30S subunit degradation intermediates can be seen on the sucrose gradients further confirms the unbalanced degradation of the ribosome subunits.

Total RNA offers a crude but effective way to estimate ribosome amount in cells, as 80% of it is rRNA (1, 19). Previous studies have shown that ribosomes are degraded as the growth conditions of the bacterial culture become limiting (4, 16). In this study, the total RNA levels per cell are measured for 14 days in stationary phase. Total RNA starts to decrease in late log and early stationary phase (Fig. S4 at http://dx.doi.org/10.23673/re-310), which is in good agreement with previously reported data (15). In this study, we see a continuous decrease in total RNA after reaching stationary phase. As stationary phase progresses, the RNA quantity continues to decrease until day 6, when approximately 65% of the total RNA is degraded (Fig. 3). After day 6, no statistically significant decrease in RNA concentration is detected.

Despite extensive ribosome degradation, r-protein quantity in the remaining ribosomes is stable over the course of 14 days in stationary phase (Fig. 4). Two r-proteins, bS1 and bS21, exhibit significant reductions in the 70S ribosomes (Fig. 4). After 12 days of cultivation, about 50% of the ribosomes have lost bS1, while bS21 is missing from 25% of 70S ribosomes (Fig. 4). Both bS1 and bS21 are important for the initiation step of translation (24, 25, 33). In E. coli, bS1 takes part in the recruitment and positioning of mRNA to the 30S subunit to form the initiation complex, and it is a part of the translating ribosomes (34). bS1 has been shown to be present at substoichiometric amounts in the nontranslating ribosomes (24). Proteins bS1 and bS21 may be removed from the ribosomes as an additional mechanism of regulation of translation initiation during stationary phase. Moreover, the substoichiometric r-protein composition is an example of the ribosome heterogeneity in stationary growth phase.

Given that most r-protein stoichiometry in the 70S ribosomes does not change during stationary phase, we compare this with the r-protein stoichiometry in the total cell proteome. During the transition from exponential to stationary growth, r-proteins are detected in the total proteome in the same stoichiometry as they are found in ribosomes, i.e., one copy of each individual r-protein per ribosome (Fig. 5A). Thus, at this time point, there are no large pools of free r-proteins in the cells except for the proteins bS1 and bL7/bL12 (Fig. 5A). During the progression of the stationary phase, the equimolar content of the r-proteins is maintained in the ribosomes (Fig. 4). Our total-proteome analysis clearly demonstrates that the stoichiometry of r-proteins is lost during stationary phase and a specific set of r-proteins accumulate as a free pool. Thirty of 51 analyzed r-proteins are found to be stable in the course of a 14-day timescale, while 21 r-proteins are subject to faster degradation (Fig. 5B). Degradation of these r-proteins occurs concomitantly with the diminishing of rRNA, although with a small delay (Fig. 6).

It is evident that r-proteins are stable as long as they are an integral part of the ribosome and become a potential substrate for the degradation machinery after the rRNA is degraded. Considering the differences in the accumulation of the free ribosomal subunits, one would expect the 30S subunit proteins to have higher degradation rates. However, according to our experimental data, both subunits contain r-proteins belonging to stable and short-lived r-proteins roughly to the same extent. Most of the smaller-sized r-proteins belong to the short-lived group, while larger-sized r-proteins tend to be in the stable group. (Fig. 7). A notable exception is the largest r-protein of the 50S subunit, uL2, which is degraded during stationary phase. The proteins, which are known to form a significant free pool already in exponential phase (bS1 and bL12), are as stable in our study (28, 29). The proteins with known nonribosomal functions (bS1, uS10, and uL4) (6) are expectedly detected as stable (Fig. 5B). The physiological significance of the other stable r-proteins remains elusive. We cannot exclude the possibility that stable r-proteins have an unknown function(s) during stationary phase. However, it is highly possible that their stability is not a question of function but an exception in regard to their structure or conformation in stationary phase. Previous studies have shown that after rRNA degradation, unbound r-proteins can reintegrate into new ribosomes (35). When a cell culture transitions into a new lag phase, these stable r-proteins can potentially be incorporated into newly synthesized ribosomes, allowing faster adaptation of bacterial growth in response to the environmental changes.

In this study, we determine that some r-proteins are not degraded concomitantly with the rRNA, but instead form a free pool of r-proteins in the cell (Table S7 at http://dx.doi.org/10.23673/re-310). Previously, the sizes of free pools of r-proteins have been determined in the cell under exponential growth conditions (29). Comparing our results characterizing stationary growth phase to exponential growth phase, the sizes of free pools of r-proteins are 20 to 2,000 times larger in stationary phase. For example, Chen et al. report that the free pool size of uL10 is 0.015 ± 0.001 per ribosome (29). However, in stationary phase, the free pool size of uL10 is 1.750 ± 0.533 per ribosome. There are also differences in the proteins that form free pools in exponential or stationary phase (Table S7 at the URL mentioned above). We conclude that the sizes of free pools of r-proteins differ both in quantity and quality between exponential and stationary-phase cell cultures.

The fact that r-proteins are present in equimolar quantities makes them a useful internal standard for proteomic analysis of bacteria. However, it should be considered that during stationary phase, the stoichiometry between individual r-proteins in the cell proteome is lost.

Based on the results reported in this paper, we propose the following model of r-protein dynamics in stationary phase. The RNA content per cell decreases by 65% from day 1 to 6. During the early stages of stationary phase, free 30S subunits might be degraded faster than free 50S subunits. The latter are degraded predominantly during the later stages of stationary phase. Twenty-one r-proteins are degraded in the stationary phase alongside rRNA, while 30 r-proteins remain in the cell as a free pool. The stability and potential functionality of these stable r-proteins is an intriguing new aspect of r-proteins and their part in cellular systems.

## MATERIALS AND METHODS

### Cell growth.

E. coli MG1655-SILAC (F*^−^ λ^−^ rph-1 ΔlysA ΔargA*) was grown in MOPS medium (36) supplemented with 0.1 mg/mL heavy-labeled arginine (Arg10, [^13^C]_6_H_14_[^15^N]_4_O_2_) and lysine (Lys8, [^13^C]_6_H_14_[^15^N]_2_O_2_) (Silantes, Germany). At mid-log phase (*A*_600_ ≈ 1), amounts of 2 mg/mL of unlabeled arginine (Arg0) and lysine (Lys0) were added to the culture. The cell culture was divided into 8 separate batches, and growth was continued for a maximum of 14 days. Cells were harvested by low-speed centrifugation (4,500 × *g*/15 min) after 24 and 48 h (from here on referred to as day 1 and day 2) of growth and subsequently on days 4, 6, 8, 10, 12, and 14. The experiment was carried out in triplicate.

As an internal reference, E. coli MG1655-SILAC cells were grown in MOPS medium supplemented with 0.1 mg/mL medium-heavy-labeled arginine (Arg6; [^13^C]_6_H_14_N_4_O_2_) and lysine (Lys4; C_6_H_10_[^2^H]_4_N_2_O_2_) (Silantes, Germany). Cells were grown to mid-log phase (*A*_600_ ≈ 1) and harvested by low-speed centrifugation (4,500 × *g*/15 min).

### Total RNA analysis.

Total RNA was extracted from 2 mg of wet cell mass (from stationary-phase culture) using hot-phenol extraction. Cells were suspended in 200 μL of buffer A (0.5% SDS and 10 mM EDTA). An amount of 200 μL of phenol/H_2_O (pH 5.5) was added to the cell suspension, mixed, and incubated at 65°C for 30 min. The sample was centrifuged (16,000 × g/10 min), and 150 μL of the water phase was transferred to a new tube. An amount of 150 μL of buffer A was added to the remaining phenol mixture, mixed, and centrifuged (16,000 × g/10 min). An amount of 150 μL of water phase was again moved to a new tube (total of 300 μL). Then, 400 μL of chloroform was added and mixed, and the phases were separated by centrifugation (16,000 × g/10 min). An amount of 200 μL of the water phase was transferred to a new tube, and RNA was sedimented by adding 5 volumes of 96% ethanol and 0.3 M sodium acetate (pH 5.5) and incubating at −20°C overnight. The precipitate was collected by centrifugation (16,000 × *g*/10 min), washed 2 times with 96% ethanol, and dried at 37°C for 5 min. The RNA precipitate was dissolved in water, and the absorbance at 260 nm (*A*_260_) was measured. RNA concentrations (*A*_260_) were normalized by dividing by the day 1 *A*_260_ value (day *n*/day 1). Total RNA concentration values were analyzed across all time points using the two-way analysis of variance (ANOVA) statistical test in GraphPad 7.0 software, and the statistical values can be found in Table S2 posted at http://dx.doi.org/10.23673/re-310. Total-RNA-concentration values were also fitted into the one-phase decay model in GraphPad 7.0 software. The one-phase decay model is as follows: *y* = (*y*_0_ − plateau) × exp(−*k* × *x*) + plateau, where *x* is time, *y* starts at *y*_0_ and then decays down to a plateau with one phase, and *k* is the rate constant in units that are the reciprocal of the *x* axis units. For our data, the plateau and *k* values were restricted to being larger than zero.

### Sucrose gradient centrifugation.

Cell pellets were suspended in lysis buffer (20 mM Tris [pH 7.5], 100 mM NH_4_Cl, 10 mM Mg-acetate, and 6 mM β-mercaptoethanol). After the addition of DNase I (40 units/mL), the cells were disrupted with glass beads using the Precellys 24 homogenizer (6,000 rpm, 4°C, 3 times for 1 min with a 1-min pause). The lysate was cleared of cell debris by centrifugation (16,000 × *g*, 20 min at 4°C). A maximum of 100 *A*_260_ units (for precise amounts, see Table S8 at http://dx.doi.org/10.23673/re-310) of supernatant was loaded onto a 15-to-25% sucrose gradient in OV-10 buffer (20 mM Tris [pH 7.5], 100 mM NH_4_Cl, 0.25 mM EDTA, and 6 mM β-mercaptoethanol) supplemented with 10 mM Mg-acetate and centrifuged at 56,000 × *g* for 16 h in a Beckman SW-28 rotor. Ribosome profiles were recorded at 260 nm. Areas under the 70S, 50S, and 30S peaks were quantified by using ImageJ, and the corresponding ratios were calculated. Subunit ratios were analyzed across all time points using the two-way ANOVA statistical test in GraphPad 7.0 software, and the statistical values can be found in Table S1 at the URL mentioned above. Fractions containing 70S ribosomes were collected for further analysis via liquid chromatography-tandem mass spectrometry (LC-MS/MS).

### Ribosome r-protein content analysis.

70S ribosomes from stationary-phase and reference cells were mixed in a 1:1 molar ratio and precipitated with 10% trichloroacetic acid (TCA) overnight at 4°C. The precipitated proteins were pelleted by centrifugation (16,000 × *g* for 60 min) at 4°C, washed twice with 80% ice-cold acetone, and air dried at 37°C for 5 min. All subsequent sample preparations were conducted at room temperature. Proteins were dissolved in 50 μL of 8 M urea/2 M thiourea solution, reduced for 1 h at 56°C by adding 1 mM dithiothreitol (DTT), and carbamidomethylated with 5 mM chloroacetamide for 1 h in the dark. Proteins were digested with endoproteinase Lys-C (Wako) at a 1:50 enzyme-to-protein ratio for 4 h. The urea concentration in the solution was reduced by adding 4 volumes of 100 mM ammonium bicarbonate (ABC), and peptides were further digested using mass spectrometry-grade trypsin (1:50 enzyme-to-protein ratio) overnight. The enzymes were inactivated by the addition of trifluoroacetic acid (TFA) to a 1% final concentration. For LC-MS/MS analysis, peptides were desalted on self-made reverse-phase C_18_ stop-and-go-extraction tip (STAGEtip) columns and analyzed by LC-MS/MS using the LTQ Orbitrap XL mass spectrometer (Thermo Scientific) coupled with an Agilent 1200 nanoflow LC system via a nanoelectrospray ion source (Proxeon). An amount of 1 mg of purified peptide was injected at a flow rate of 700 nl/min into a 75-mm by 150-mm fused silica emitter (Proxeon) that was packed in-house with 3-mm ReproSil-Pur 120 C_18_-AQ stationary-phase beads (Dr. Maisch GmbH) and eluted over 120 min using a linear gradient of 3% to 40% solvent B (80% acetonitrile and 0.5% acetic acid) in solvent A (0.5% acetic acid) at a flow rate of 250 nl/min. The LTQ Orbitrap XL mass spectrometer was operated in a data-dependent mode, and a “lock mass” option was enabled for *m/z* 445.120030 to improve mass accuracy. Precursor ion full-scan spectra (*m/z* 300 to 1,800) were acquired in the Orbitrap in profile mode with a resolution of 60,000 at *m/z* 400 (target value of 1,000,000 ions and maximum injection time of 500 ms). The five most intense ions were fragmented in the linear ion trap by collision-induced dissociation (normalized collision energy, 35.0%), and spectra were acquired in centroid mode (target value of 5,000 ions and maximum injection time of 150 ms). The dynamic exclusion option was enabled (exclusion duration of 120 s), and ions with an unassigned charge state, as well as singly charged ions, were rejected. The number of peptides per protein analyzed is shown in Table S9 at http://dx.doi.org/10.23673/re-310.

### Total-proteome analysis.

Cells were suspended in 10 volumes of 4% SDS, 100 mM Tris‐HCl, pH 7.5, 100 mM DTT lysis buffer. Cell suspensions were heated at 95°C for 5 min and lysed by sonication (Bandelin) (60 1-s pulses at 50% intensity). Cell debris was removed by centrifugation at 14,000 × *g* for 10 min. The protein concentration was determined at *A*_280_ using bovine serum albumin (BSA) as a standard. An amount of 7.5 μg of total protein from stationary-phase cell lysates was mixed in a 1:1 ratio with total protein from reference cell lysates. For r-protein stoichiometry in the early-stationary-phase total proteome, 18 μg of total protein from stationary-phase cell lysates was mixed in a 24:1 ratio with 70S ribosomes (0.763 μg). Samples were precipitated with 2:1:3 methanol/chloroform/water. Protein pellets were suspended in 25 μL of 7 M urea, 2 M thiourea, followed by disulfide reduction with 5 mM DTT for 30 min and cysteine alkylation with 10 mM chloroacetamide for 30 min at room temperature. Proteins were digested with endoproteinase Lys-C (Wako) at a 1:50 enzyme-to-protein ratio for 4 h. The urea concentration in the solution was reduced by adding 4 volumes of 100 mM ABC, and peptides were further digested using mass spectrometry-grade trypsin (1:50 enzyme-to-protein ratio) overnight. Enzymes were inactivated by the addition of TFA to a 1% final concentration. Peptides were desalted with self-made reverse-phase C_18_ STAGEtip columns. The resulting peptides (Table S10 at http://dx.doi.org/10.23673/re-310) were fractionated and analyzed by LC-MS/MS (37).

### Mass spectrometry data analysis.

Data analysis was performed using MaxQuant (version 1.5.6.0) with default settings (38), except that the minimal peptide length for the specific and nonspecific search was 5 amino acids. Unique peptides were used for quantification, the main search peptide tolerance was 8 ppm, and variable modification was used for the quantitation of oxidation (methionine). The peptide identification search was carried out against the E. coli K-12 MG1655 protein sequence database from UniProtKB (as of October 2019). The search results were filtered and transformed using Perseus (version 1.6.14.0) (39). For proteins bL20, bL33, bL34, bS20, and bS21, MS data analysis was done using the Mascot search engine and Skyline as described in reference 22. Each protein was quantified through H/M, L/M, and/or (L+H)/M SILAC ratios, comparing the relative unlabeled (L) and/or heavy-labeled (H) quantities against the medium-heavy-labeled (M) internal reference quantities. Proteins bL31A, bL31B, bL35, bL36A, and bL36B could not be quantified reproducibly in our data sets due to small numbers of unique peptides. H/M, L/M, and (L+H)/M values were analyzed across all time points using the two-way ANOVA statistical test in GraphPad 7.0 software, and the statistical values can be found in Tables S4 to S6 at http://dx.doi.org/10.23673/re-310.

### r-protein stability modeling in total-proteome data sets.

Individual r-protein degradation dynamics were analyzed using nonlinear regression in GraphPad 7.0 software. Data were fitted into plateau followed by one phase decay model: IF *x* < *x*_0_, THEN *y* = *y*_0_, ELSE *y* = Plateau + (*y*_0_ − Plateau) × exp(−*k* × (*x* − *x*_0_)), where *x* is time, *y* is *y*_0_ until *x* < *x*_0_ and then decays down to a plateau with one phase, and *k* is the rate constant in units that are the reciprocal of the *x*-axis units. For our data, the plateau and *k* values were restricted to being larger than zero. The alternative model was a straight line, as follows: *y* = *y*-intercept + slope × *x*. Data were weighted by 1/*y*^2^; this minimized the sum of the squares of the relative distances of points from the curve. The fit of the models was compared using Akaike’s information criterion (30), and the statistical values can be found in Table S6 at http://dx.doi.org/10.23673/re-310.

### Data availability.

Proteomics data can be found in the EMBL-EBI Proteomics Identification database (PRIDE). Extended supplemental materials can be found in the DataDOI database at http://dx.doi.org/10.23673/re-310.
